# The commensal infant gut meta-mobilome as a potential reservoir for persistent multidrug resistance integrons

**DOI:** 10.1038/srep15317

**Published:** 2015-10-28

**Authors:** Anuradha Ravi, Ekaterina Avershina, Steven L. Foley, Jane Ludvigsen, Ola Storrø, Torbjørn Øien, Roar Johnsen, Anne L. McCartney, Trine M. L’Abée-Lund, Knut Rudi

**Affiliations:** 1 Norwegian University of Life Sciences, Chemistry, Biotechnology and Food science department (IKBM), Campus Ås, Ås 1432, Norway; 2 National Center for Toxicological Research, U.S. Food and Drug Administration, Division of Microbiology, Jefferson, AR 72079; 3 Department of Public Health and General Practice, Norwegian University of Science and Technology, 9491 Trondheim, Norway; 4 Microbial Ecology & Health Group, Department of Food and Nutritional Sciences, University of Reading, Reading, UK; 5 Norwegian University of Life Sciences, Department of Food safety and Infection Biology, Campus Adamstuen, Oslo 0454, Norway

## Abstract

Despite the accumulating knowledge on the development and establishment of the gut microbiota, its role as a reservoir for multidrug resistance is not well understood. This study investigated the prevalence and persistence patterns of an integrase gene (*int1*), used as a proxy for integrons (which often carry multiple antimicrobial resistance genes), in the fecal microbiota of 147 mothers and their children sampled longitudinally from birth to 2 years. The study showed the *int1* gene was detected in 15% of the study population, and apparently more persistent than the microbial community structure itself. We found *int1* to be persistent throughout the first two years of life, as well as between mothers and their 2-year-old children. Metagenome sequencing revealed integrons in the gut meta-mobilome that were associated with plasmids and multidrug resistance. In conclusion, the persistent nature of integrons in the infant gut microbiota makes it a potential reservoir of mobile multidrug resistance.

The spread of antibiotic resistance (AR) genes and development of multidrug resistance represent major threats to public health^1^. Until recently, pathogens have been the prime focus with respect to understanding the spread of multidrug resistance, with the commensal microbiota receiving much less attention. However, recent studies have shown the prevalence of AR genes in the commensal gut microbiota^2^,^3^,^4^,^5^. Furthermore, the gut microbiota shows a high rate of horizontal gene transfer (HGT), which was indicated to be up to 25-fold greater than that of bacteria in other environments^6^. Hence, the collective mobile genetic elements (MGEs) in the gut microbiota (i.e. the gut meta-mobilome) represent an important target for both understanding and combating the spread of multidrug resistance^5^,^7^.

The gut microbiota forms a complex ecosystem. The gut is assumed sterile at birth^8^,^9^ whereas just after birth, it goes through major shifts starting with facultative anaerobic bacteria (*Enterococcaceae* and *Streptococcaceae*)^10^,^11^. As oxygen levels deplete, strictly anaerobic bacteria (*Bifidobacteriales* and *Bacteroidetes*) take over and dominate in the gut^12^. This progression slows down as the microbiota reaches the adult-like state where an estimated 100–200 species co-exist in close proximity^13^. Although scientists have started to understand the shifts in the taxonomic composition of the developing microbiota from infancy to adulthood, the knowledge of the meta-mobilome, including the transmission and persistence of multiple antimicrobial resistance genes, is limited.

Antimicrobial resistance genes can be carried in integrons, which are non-mobile elements themselves, but are often found within MGEs like transposons and plasmids^14^,^15^. Integrons are platforms for integration, assembly and expression of specific gene cassettes within the MGEs that often encode antimicrobial resistance^16^. The individual genetic cassettes typically lack their own promoters, but are expressed by a common promoter for all the cassettes within the integron (Fig. 1). There have been 5 classes of integrons (class I–V) classified to date^5^. The class I integrons are the most widely studied and are found in a broad host range of commensal and pathogenic bacteria^17^. Class I integrons are found extensively in clinical isolates containing several different AR gene cassettes conferring resistance to antibiotics commonly used against bacterial infections^16^,^18^. Up to 8 gene cassettes have been found in a single class I integron^16^, however hundreds of gene cassettes have been detected in so-called super-integrons^19^.

The aim of the current study was to investigate the prevalence and persistence of class I integrons in a large unselected longitudinal cohort of mothers and their children. We used quantitative PCR to identify and study the persistence patterns of integrons. 16S rRNA and metagenome deep sequencing were used to analyze the phylogeny and genetic background of the integrons in the samples and to trace these elements longitudinally.

## Materials and Methods

**Figure 2:**
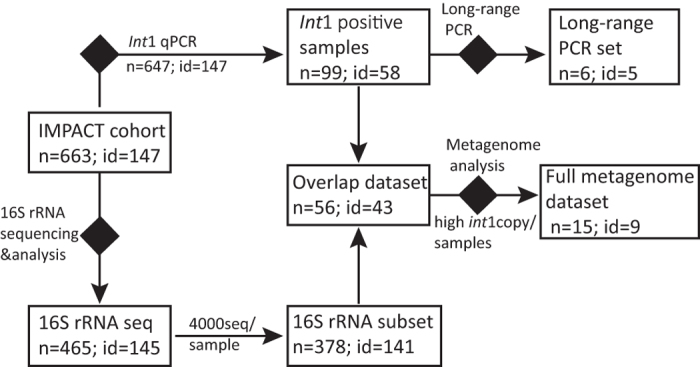
Workflow of experimental setup.

The schematic overview of the workflow is displayed in Fig. 2. The methods were performed in accordance to the approved guidelines and all experimental protocols were approved by Norwegian University of Life Sciences.

### Cohort description

IMPACT (Immunology and Microbiology in Prevention of Allergy among Children in Trondheim) study is a controlled non-randomized longitudinal study, which began in 2000. The regional committee for Medical Research Ethics for Central Norway has approved the IMPACT study (ref. 120–2000). This study was granted a license by the Norwegian Data Inspectorate to process personal health data and one of the parents of each child signed a written informed consent form (r. 2003/953-3 KBE/-). Current controlled trials registration number: ISRCTN28090297.

The study involved 720 pairs of pregnant women and their children (up to two years of age). Ninety percent of the children were vaginally delivered and at term. Ninety-seven percent of the infants were breast-fed exclusively for the first six weeks of life. Fecal samples were collected from the pregnant women during the first/second (7–20 weeks) trimester and the third (32–40 weeks) trimester, and from the children at 3–10 days, 4 months, 1 and 2 years of age. In the current study, samples from a randomly selected subgroup of 147 mother-child pairs from the IMPACT cohort were analyzed. Information on allergy related hereditary diseases, atopy and antibiotic usage; health and exposure factors for the parent and child is summarized in Supplementary Table S1.

### Sample collection

Fecal samples from the subjects of the IMPACT cohort were collected in Cary-Blaire transport and holding medium (BD Diagnostics, Sparks, MD). The samples were frozen at -20 °C within 2 h from collection. The samples were then stored at −80 °C within one month for children and mothers.

### DNA purification

Fecal DNA was purified with an automated protocol using DNA extraction kit based on paramagnetic particles (LGC Genomics, UK). In brief, the samples were subjected to mechanical lysis using glass beads and the DNA was purified by eluting from the paramagnetic particles by downstream processes as described by manufacturer. The DNA was stored at −40 °C.

### Gene quantification

The abundance of integrons (using the integrase (*int1*) gene^20^ as a proxy) in the samples was calculated relative to the 16S rRNA gene by quantitative PCR. Each PCR reaction (25 μl) contained 1× HOT FIREPol PCR mix (Solis BioDyne, Estonia); 200 nM forward and reverse primers; one μl of sample DNA and water. The reaction mix was run on LightCycler 480 (Roche, Germany). Following the thermal cycling the raw fluorescence data was exported into LinRegPCR program. The software performed baseline corrections and calculated the mean PCR efficiency. For the *int1* amplicon, we also used High Resolution Melting (HRM) curve analysis, in addition to Sanger sequencing using the BigDye Terminator v.1.1 chemistry (Applied Biosystems) for verification.

The thermal cycling for the 16S rRNA primer-pair (5′-TCCTACGGGAGGCAGCAGT-3′; 5′- GGACTACCAGGGTATCTAATCCTGTT-3′) was an initial denaturation of 95 °C for 15 min followed by 40 cycles of 95 °C for 30 sec and 60 °C for 30 sec. This primer-pair targets conserved regions of the 16S rRNA gene^21^. The primers flanking the *int1* gene (5′-ACGAGCGCAAGGTTTCGGT-3′; 5′-GAAAGGTCTGGTCATACATG-3′) from Sørum *et al.*^11^ were used with thermal cycling conditions 95 °C for 15 min and 40 cycles of 95 °C for 30 sec, 53 °C for 30 sec and 72 °C for 30 sec.

### Microbial community analyses

Microbial communities were assessed using Illumina sequencing of 16S rRNA gene amplicons (n = 465), with subsets subjected to full metagenome (n = 15) and long-range PCR amplicon (n = 6) analyses. For full metagenomics analysis, samples were selected based on the high relative quantities of *int1* gene in the samples. For a long-range PCR, six *int1*-positive samples were randomly chosen for amplification.

Long-range primers were used to amplify the sequence flanking the region from *attI* to the 3′ consensus region including the gene cassettes (5′-GGCATCCAAGCAGCAAG-3′; 5′-AAGCAGACTTGACCTGA-3′)^11^ with the TaKaRa LA PCR kit Ver.2.1. The thermal cycling conditions of 94 °C for 5 min followed by 35 cycles of 98 °C for 10 min, 54 °C for 30 sec and 72 °C for 1 min, with the final extension step at 72 °C for 5 min. The resultant PCR products were analyzed by agarose gel electrophoresis and Illumina sequencing.

For full metagenome, long-range amplicon and metagenome analyses, gDNA was randomly fragmented, tagged, amplified and prepared for sequencing using Nextera XT kit (Illumina, USA).

Portions of the 16S rRNA genes were amplified using PRK341F/PRK806R primers targeting V3-V4 regions^22^, modified by addition of Illumina-specific adapters. Each PCR reaction (25 μl) contained 1× HOT FIREPol PCR mix (Solis BioDyne, Estonia); 200 nM uniquely tagged forward and reverse primers; 1 μl of sample DNA and water. The thermal cycling conditions were 95 °C for 15 min and 30 cycles of 95 °C for 30 sec, 50 °C for 1 min and 72 °C for 45 sec. PCR products were then pooled, based on their concentrations measured using Quant-iT^TM^ PicoGreen® dsDNA assay kit (Life Technologies, USA), column-purified using E.Z.N.A.® Cycle Pure kit (Omega Bio-tek, USA) and submitted for sequencing.

Sequencing was performed on MiSeq platform (Illumina, USA) using V3 sequencing chemistry with 300 bp paired-end reads. 16S rRNA gene amplicon samples were processed at Norwegian Sequencing Centre (Oslo, Norway), whereas full metagenome samples were sequenced in-house.

### Bacterial culturing

For isolation of *Bifidobacterium* species, 10-fold dilutions of fecal samples in 1% peptone water were anaerobically cultured on Beerens agar at 37 °C. Isolated colonies were then subcultured to purity using the same conditions. DNA was extracted for sequencing of 16S rRNA gene as described above to confirm isolates belonging to *Bifidobacterium* genus.

Three-fold serial dilutions of fecal samples from the cohort were prepared in distilled water, cultured on lactose agar and in tryptic soya broth with 5% horse blood, incubated at 37 °C for 24 h. The broth was supplemented with 0.1% of both Tween 80 and magnesium chloride to recover damaged *Enterobacteriaceae* cells.

### Data analyses

16S rRNA gene amplicon data were analyzed using QIIME pipeline^23^. Sequences were first quality filtered (*split_libraries.py*; sequence length between 200 bp and 1000 bp; minimum average quality score 25; not more than 6 ambiguous bases; and no primer mismatch allowed) and then clustered at 99% homology level using closed-reference *uclust* search against Greengenes database^24^ (*pick_closed_reference_otus.py*). Persistence of operational taxonomic units (OTUs) over time in individuals was assessed using multi-way decomposition PARAFAC analysis of mean-centered abundance data^25^. This analysis allows detection of the OTUs that bring most of the variation into the system, simultaneously with detecting the time points at which these OTUs are most pronounced. Simpson’s reciprocal diversity index and Bray-Curtis dissimilarity index were used for alpha- and beta-diversity assessment, respectively.

Metagenome data mapping and assembly was performed using Geneious pipeline following authors’ recommendations^26^. MG-RAST metagenome analyzer was used to analyze the taxonomy and functional classification of the samples^27^. PATRIC database^28^ in MG-RAST was used to check the integron abundance in the samples. E-value < 10^−5^ was used as the cut-off to select integron hits.

*Int1* gene persistence was calculated as the ratio of the number of mother-child pairs in who *int1* was detected at both time points to the total number of mother-child pairs for who information for both time points was available. The odds ratio for *int1* gene detection was calculated by the ratio of *int1* persistence to the prevalence of *int1* at a later time point.

Fisher exact test, Pearson correlation coefficient and Spearman correlation coefficient were used for pairwise comparisons of *int1* and 16S rRNA data (including diversity, OTU abundance and bacterial class abundance data). The significance of the change over time was tested with Friedman’s test - a non-parametric version of ANOVA test which takes into account repeated measurements. The change in *int1* gene relative abundance was also compared to the change in log-transformed OTU relative abundances over time in an attempt to identify OTUs that correlated to *int1*. Regression and classification decision trees were also built in an attempt to identify bacterial classes that correlated to *int1*. Data analyses were performed using MATLAB® R2014a software (The MathWorks Inc., Natick MA, USA).

## Results

### Microbiota composition and development

The phylogenetic composition of the microbiota was assessed using deep 16S rRNA gene sequencing. All samples that were amplified with 16S rRNA gene-targeting primers and further amplified with Illumina-adapted primer set were included in the analysis. In total, sequencing data were available for 451 samples. In addition, seven of the samples were analyzed in triplicate to determine technical variation, which was found to be low (Supplementary Fig. S1). The average quality score for the sequence range of 250–299 bp was 25.

**Figure 3:**
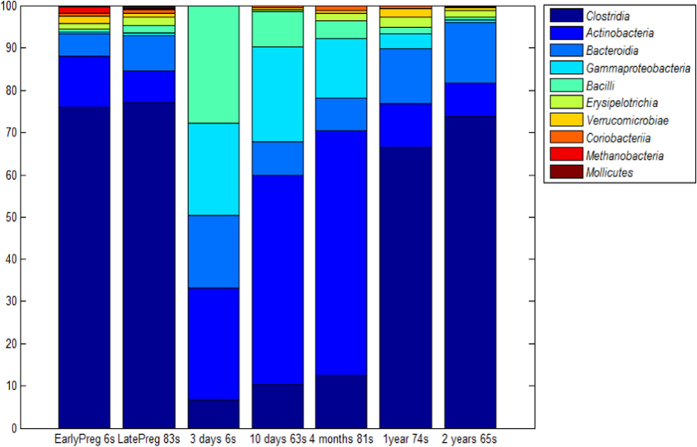
Bacterial class composition of stool samples of infants (from 3 days to 2 years of age) and their mothers during early (1/2 trimester; EarlyPreg) and late (final trimester; LatePreg) pregnancy based on the deep sequencing of 16S rRNA gene amplicons. s, Number of samples at each time period.

**Figure 4:**
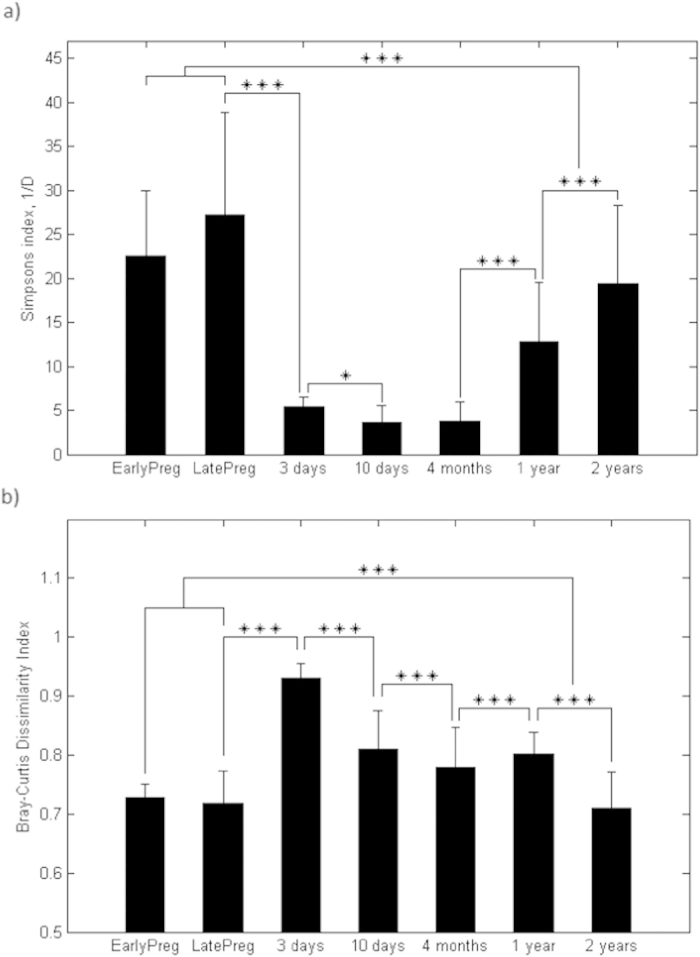
Diversity characteristics of stool samples of infants (from 3 days to 2 years of age) and their mothers during early (1/2 trimester; EarlyPreg) and late (final trimester; LatePreg) pregnancy based on the deep sequencing of 16S rRNA gene amplicons. (**a**) Simpson’s reciprocal index of alpha-diversity. (**b**) Bray-Curtis dissimilarity index of beta-diversity. *p value < 0.05; and ***p value < 0.001.

On average, 21,277 sequences per sample were generated after quality filtering and assembly. To ensure even amount of sequencing information, 4,000 reads per sample were randomly picked from the full dataset based on the recommendations by Sørensen *et al.*^29^. The final dataset after quality filtering and unification of the sequencing information per sample comprised 378 samples, with a total of 8,288 OTUs belonging to 27 classes. The 10 most abundant classes comprised nearly 100% of the microbiota at all ages (Fig. 3). Stool samples from newborns and 4-month-old infants were significantly lower in alpha-diversity and significantly higher in beta-diversity than stool samples from 2-year-olds and their mothers (Fig. 4). At 1 year of age, both alpha- and beta-diversity estimates were significantly higher than that of 4 month-olds. There was a high dominance of *Clostridia* in stool samples from mothers, as well as from 1- and 2-year-olds. Five bacterial classes were relatively equal in abundance in neonatal stool samples collected soon after birth (3 days), whereas *Actinobacteria* became dominant thereafter (4–10 days) and remained so through at least first 4 months of age. By 1 year of age, the average profile of stool samples from children had started converging towards the adult profile. However, pronounced differences in the abundance of *Actinobacteria* and *Bacteroidia* were seen between adults and 2-year-old children, suggesting climax adult community was still not reached by 2 years of age.

### Microbiota persistence and stability

**Figure 5:**
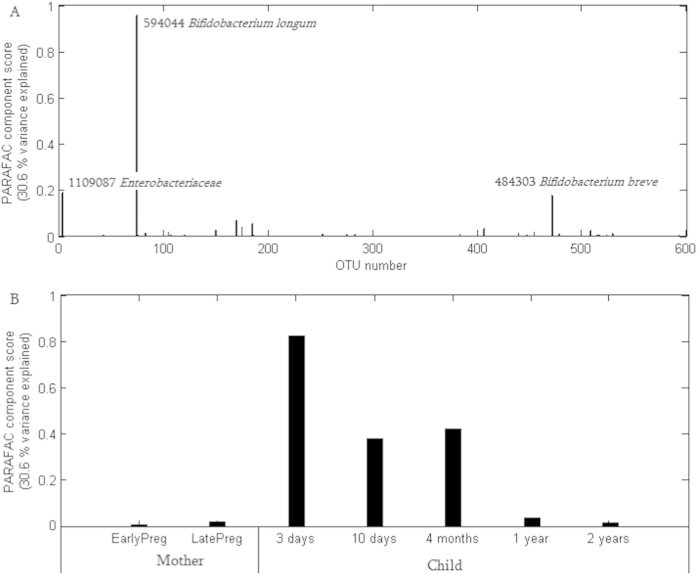
Summary of PARAFAC analysis (multi-way decomposition which defines most influential OTUs over time) on the relative abundances of 599 most abundant bacterial OTUs. (**A**) Most influential OTUs. (**B**) Time points associated with most influential OTUs.

The persistence of 599 most abundant OTUs in the dataset (with an abundance level ≥0.5% in at least one sample) were analyzed using PARAFAC. No significant associations of OTUs to age were identified when only considering the detected/non-detected information. When abundance levels were considered, two OTUs belonging to *Bifidobacterium* species (*B. longum* OTU594044 and *B. breve* OTU484303), and one assigned to *Enterobacteriaceae* family (OTU1109087), showed highest stability over time in the cohort (Fig. 5). Spearman correlation test identified the persistence of the *B. longum*-assigned OTU, which had a highest loading in PARAFAC, from 3–10 days to 4 months of age (correlation coefficient = 0.49; p = 0.007). The two other OTUs, however, did not show any significant correlations between the age groups.

### Integron distribution and persistence

**Figure 6:**
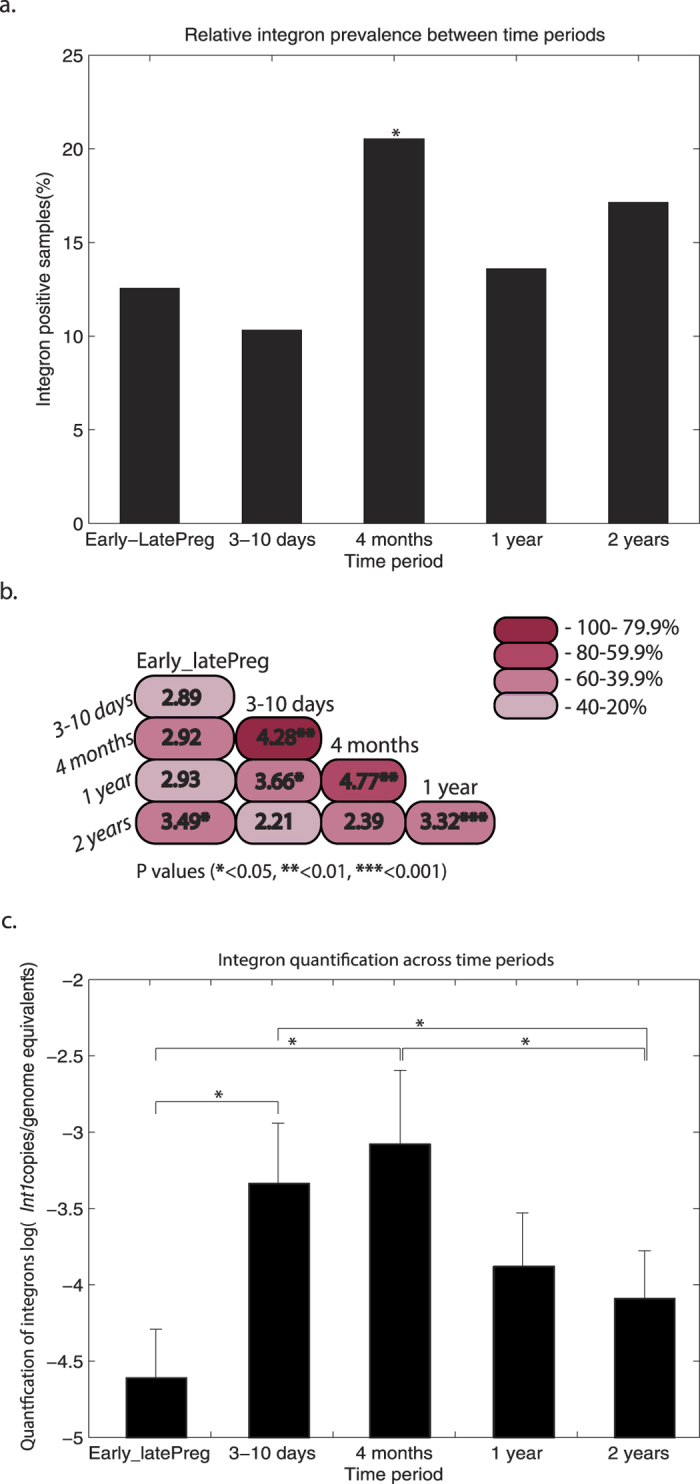
Prevalence, persistence of integron-positive samples and relative quantity of integrons in the positive samples between time points. (**a**) Relative prevalence of integron-positive samples in the dataset. *Binomial testing between the highest abundance (4 months) and the rest (p value = 0.005). (**b)** Persistence of integrons at each time point. The numbers represent the odds-ratio; the color gradient represents the percentage of persistence between time points. Significant p values by Fisher exact test are also indicated (*p value < 0.05; **p value < 0.01; ***p value < 0.001). (**c)** Relative integron quantification at each time point (log (*int*1 copies/genome equivalent^1^) for integron-positive samples. Error bars represent standard error of the mean (SEM). The significant difference between sample groups was calculated by Kruskal-Wallis test; p value < 0.05 is indicated by bracketing. Early_latePreg, samples collected from mothers during early (7–20 weeks) and late (32–40 weeks) pregnancy; 3–10 days, samples from 3- to 10-day-old infants; 4 months, 1 year and 2 years; samples from 4-month-old infants, 1-year-old and 2-year-old children, respectively. ^1^16S rRNA copies of all samples from different age groups were normalized to reflect genome equivalents taking into account copy number information given by Vetrovsky *et al.*^30^.

The distribution of integrons was analyzed by quantitative PCR of the *int1* gene. All samples were included and amplification was controlled by 16S rRNA gene amplification. Out of initial 663 IMPACT samples, 16 failed to amplify PCR products using 16S rRNA gene-targeting primers and thus were excluded from the analysis. In total, 99 of the 647 samples analyzed showed the presence of integrons. The prevalence of the integron-positive samples was highest from 4-month-old children compared to any other age (Fig. 6a). The highest persistence patterns for integrons were seen in children between 3–10 days and 4 months, and 4 months to 1 year (Fig. 6b). Persistence between some mother-child pairs was also detected. The *int1* gene copy numbers of the positive samples, corrected for the estimated genome equivalents^30^, were significantly higher in samples from infants (3–10 days and 4 months) compared to both pregnant mothers and 2-year-old children (Fig. 6c).

For the children with persistent *int1* genes, 17% (1 of 6 children with antibiotic usage information) received antibiotics during the first year of life. In addition, 31% (46 out of 147) of the children in the whole cohort had antibiotic usage information documented.

### Correlation of *int1* gene to 16S rRNA gene

Detection of *int1* gene did not correlate to alpha-diversity (Simpson’s reciprocal index 1/D = 12.3 ± 1.74 [mean ± SEM] and 1/D = 13.7 ± 0.66 for *int1*-positive and *int1*-negative subgroups, respectively) or to beta-diversity (Bray-Curtis Dissimilarity index BC = 0.85 ± 0.03 and BC = 0.86 ± 0.04 for *int1*-positive and *int1*-negative subgroups, respectively). There was also no significant correlation detected between alpha-diversity and *int1* gene relative abundance (correlation coefficient = −0.389, p = 0.45).

With respect to OTU quantity, the most persistent OTU (*B. longum* OTU594044) showed a positive correlation with the *int1* gene (p = 0.03) at 3–10 days. No other significant correlations, however, were found (Supplementary Fig. S2). Additionally, it was investigated whether a change in OTU relative abundance could be associated to the change in *int1* gene relative abundance over time, but there was not an OTU identified that was significantly associated to the *int1* gene. Finally, the analyses concentrated on the OTUs that were detected in all samples for which *int1* were detected (Supplementary Table S2); however, these OTUs did not show any quantitative correlations with *int1* either.

There were additional attempts to find bacterial classes that might correlate to *int1* detection or *int1* gene abundance; however, no significant pairwise correlations between bacterial classes and *int1* gene abundance were detected (Supplementary Fig. 3). Regression and classification decision trees were then built to test for the cumulative effects of bacterial classes, but these analyses also suggested weak correlations between 16S rRNA gene and *int1* gene data (Supplementary Fig. S4 and Supplementary Fig. S5 for regression and classification, respectively).

### Search for *int1* gene in bacterial isolates

The detection of the *int1* gene in the genomes of sequenced representatives of persistent/stable OTUs identified by PARAFAC was carried out by BLAST searching whole-genome sequencing data from 16 *B. longum*, 2 *B. breve* and 10 *E. coli* strains, isolated from previously published subset of the IMPACT dataset^31^,^32^. Despite high numbers of hits to 16S rRNA gene per isolate (451.5 ± 37.8), which was used as a proxy for the genome coverage, the analyses failed to identify reads that showed homology to the *int1* gene sequence identified in our work.

### Long-range PCR and amplicon sequencing

**Figure 7:**
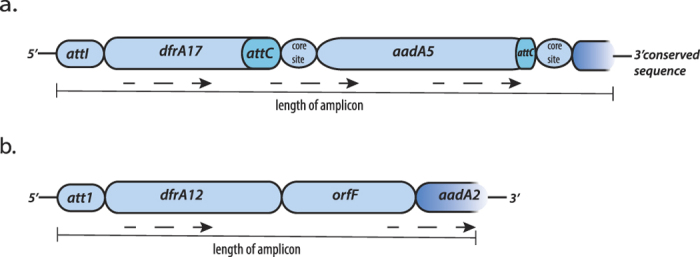
Integrons detected by long-range PCR. (**a**) 1.5 kb partially sequenced integron by long-range PCR product. (**b**) 1.1 kb partially sequenced integron by long-range PCR. Cylindrical boxes show individual genes that are size dependent, i.e. larger box is longer gene; dotted arrows indicate the direction of transcription; and gradient blue color the end of the acquired sequence. Gene and structural features: *attI*, primary recombination site; *dfrA17* and *dfrA12*, dihydrofolate reductase; *attC*, recombination site; *aadA5* and *aadA2*, aminoglycoside adenylyltransferase; and *orfF*, hypothetical protein.

Six integron-positive samples were randomly selected for long-range PCR and sequencing. The reads were assembled into contigs and two contigs of lengths 1541 bp and 1019 bp showed BLAST hits to *E. coli* strain DK510 (GQ906578.1) containing dihydrofolate reductase (*dfrA17*) and aminoglycoside adenylyltransferase (*aaDA5*) genes (E-value = 0) with 100% identity (Fig. 7a) and *E. coli* strain A30 (KF921570.1) containing dihydrofolate reductase (*dfrA12*), hypothetical protein (*orfF*) and aminoglycoside adenylyltransferase (*aadA2*) gene cassettes (E-value = 0) with 100% identity (Fig. 7b), respectively.

### Integron presence in shotgun metagenome data

Fifteen samples (late pregnancy, n = 1; 3–10 days, n = 6; 4 months, n = 5; and 2 years, n = 3) having microbiota profile information and highest relative abundance of *int1* were selected for shotgun metagenome sequencing. On average, 837,048 reads with a size range from 35 bp to 301 bp were obtained for each sample. By NCBI BLAST searches 699 shotgun metagenomic reads from 12 samples were identified that showed high homology to the *int1* gene (E-value < 10^−5^; average identity [range] 97.5% [85.1%; 100%]; average query coverage 99.7% [98.4%; 100.0%]).

Using the MG-RAST metagenome analyzer^27^, it was found that all the samples showed the presence of integrons and integron-related genes. The identity of the integron hits of the samples were obtained from PATRIC database (Supplementary Table S3).

### Metagenome assembly and identification of complete integrons

**Figure 8:**
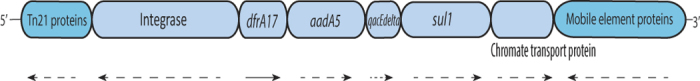
Graphical representation of a transposon-containing integron by *de novo* assembly. The boxes illustrate the coding region of genes, dark blue represents genes of the transposon and the light blue indicates genes of the integron. Genetic features: *dfrA17*, trimethoprim resistance protein; *aad*A5, streptomycin and spectinomycin resistance protein; *qacEdelta*, quaternary ammonium compound resistance protein; *sul1*, sulphonamide resistance protein.

The reads were extracted that showed *int1* homology in only one direction of the paired-end reads (n = 71) to investigate the genetic background of their paired mates. By BLAST searching of these sequences against NCBI database, candidate plasmid pSH1148_107 (GenBank JN983049) was identified that was most prevalent among the hits (Supplementary Table S4). The metagenomic reads were then mapped onto the complete plasmid sequence and approximately 60% of the plasmid was encompassed by the metagenomic reads. Seventeen of the 25 conjugation proteins of the plasmid mapped to our reads, including the *Inc1* conjugative transfer proteins, DNA primase and pilus biogene (Supplementary Fig. S6). The reads partially covered the origin of replication. There was one child who showed high prevalence of a plasmid related to pSH1148_107 (more than 1% of all reads) in stool samples from both 3–10 days and 4 months (20× and 34× mean coverage for 3–10 days and 4 months, respectively). The 3–10 days and 4 months reads mapped similarly to the plasmid. The *de novo* assembly of the reads mapped to a transposon containing integron with the *sul*1 gene and *aad*A gene cassette, which was similar to the resistance genes in pSH1148_107, and an additional *dfrA17* gene cassette (Fig. 8). The gene cassettes encode resistance to sulphonamides, spectinomycin and streptomycin, and trimethoprim respectively.

The long-range PCR amplicon contigs were also mapped to the integron assembled from our metagenome. The 1541 bp-long contig showed 97% coverage, suggesting both assemblies came from the same integron. The other contig of 1019 bp length had different gene cassettes and thus showed only partial coverage.

### Taxonomic range of the integrons identified by long-range PCR

BLAST searching of the NCBI database with the *int1*-containing contigs identified by long-range PCR revealed high homology (100% pairwise identity with 100% query coverage) towards plasmids isolated from *E. coli*, *Kluyvera georgiana*, *Salmonella enterica* and *Shigella flexneri* (Supplementary Table S5), all belonging to *Enterobacteriaceae* family.

### Search for integrons in other metagenomes

To search for the same integron in other publically available metagenomes, data was extracted from 60 metagenome samples from the cohort provided by Yatsunenko *et al.*^33^. The available cohort contained fecal samples from healthy children and adults in Malawi, United States and Venezuela; and 20 metagenomes from each of the respective countries was analyzed. Eleven (18.3%) of the metagenomes showed the presence of *int1* gene. Seven of the *int1*-positive metagenome samples also contained reads mapping to the transposon flanking regions. However, the integron-associated gene cassettes were not similar to those detected in our dataset (Supplementary Table S6).

## Discussion

Several studies have shown a high prevalence of AR genes in infants with the absence of antibiotic treatments^3^,^34^,^35^ which is in line with our findings. However, to our knowledge, this study is the first one to observe high *int1* gene prevalence and persistence. A high prevalence of integrons was found at 3–10 days and 4 months of age. In the early periods of life the resistance against colonization by exogenous bacteria is low^35^, therefore opening for the possibility of establishment of bacteria from the environment. A plausible explanation for the high integron prevalence at early age could be the hospital environment, since children are first exposed to this atmosphere^36^. There was also persistence of *int1* gene throughout the first two years of life and between mothers and their 2-year-old children, pointing towards maternal source as another potential route for transmission. Similar patterns have also been detected in transposon-associated genes in mother-infant pairs^3^,^31^. An alternative explanation, though, could be the colonization by various integrons at different ages. However, taking into account the increased likelihood of *int*1 detection at one time period given it was detected previously, the more probable explanation would be the persistence of the same integron rather than the detection of independent multiple colonization events.

The persistence of integrons in the gut microbiota indicates the versatility of MGEs to endure the drastic changes that occur during first years of life^10^,^33^. However, it is unlikely that antibiotic treatment influences the presence of multidrug resistance integrons since we did not find any alteration of persistence patterns in our dataset associated with antibiotic usage.

Diversity estimates of the cohort corresponded well with previously published observations of increase in alpha- and decrease in beta-diversity with age^11^,^33^. Interestingly, when *int1* gene abundance was highest at early days of life, the microbial diversity was lowest, suggesting that *int1* gene should be associated to those few bacteria that are established by then. However, there was no correlation between *int1* gene and diversity estimates or bacterial classes. Moreover, despite numerous attempts, we could not associate *int1* gene to any particular phylotype across individuals within our cohort. Hence, it is unlikely that the integrons have a strict phylotype association. In addition, when we tried to search for *int1* gene in *Bifidobacterium* isolates that represent the most abundant bacterial group in infancy and that was the only bacterial genus correlating to *int1* gene abundance at early infancy, we failed to find any indication of integron presence in its genomes. Lack of association between integrons and phylotypes across large phylogenetic distances has previously been observed^37^. Statistical inconsistencies have been reported when phylogenetic trees were obtained for *int1* gene and molecular marker for phylogeny such as RNA polymerase subunit B (*rpo*B)^37^. Therefore, given the broad host range for integrons^38^, the most plausible explanation for the lack of phylotype association is high rates of HGT. In concordance with potentially high HGT rates, a possible transposon carrying an integron was identified in our samples, suggesting MGEs as the likely vehicle for mobility of integrons. The mobile nature of integrons-associated MGEs has been previously observed in pathogenic bacteria^39^,^40^, environmental samples^41^ and in hospital environments^42^. We also observed the persistence of a transposon-containing integron on a potential conjugative plasmid in one infant at two time periods. This integron contained genes associated with aminoglycosides and sulfonamide resistance similar to the conjugative plasmid pSH1148_107, along with and additional trimethoprim resistance gene.

We expanded our search for integrons from different samples in our dataset by involving long-range PCRs that could amplify the whole integron. Two class I integrons were identified with potential association to a mobile element having resistance genes to trimethoprim, streptomycin and spectinomycin. Interestingly, a study by Shahcheraghi *et al.* also found a similar integron containing resistance genes in enteropathogenic *E. coli* strains (JX442969.1) isolated from fecal samples of children less than 5 years of age^43^. These evidences give further support that integrons can be reservoirs for AR genes in infants, with the potential for transmission to pathogens^4^,^35^. Additionally, we also detected integrons with different gene cassettes in publicly available metagenomes, suggesting the diversity of integrons in global human populations.

Our observation of integron-containing elements regardless of antibiotics intake suggests that they can persist without outer selection pressure. A recent study on the gut microbiota of an isolated group of Yanomani Amerindian tribe showed a similar pattern of the carriage of a pool of mobilizable next-generation antibiotic resistance genes without any prior antibiotic pressure^44^. Moreover, Stern and colleagues found over 10,000 contigs containing potential mobile elements in the MetaHIT dataset^45^, which were likely to be quite common constituents of the gut microbiota since all were identified as targets for CRISPR elements. Interestingly, only around 10% of these contigs were of viral nature, leaving the rest to plasmids and MGEs, suggesting that the host actually counter selects these mobile elements. This finding supports the selfish parasitic-like spread of conjugative plasmids associated integrons in the gut.

The overall results of the study provide evidence for high prevalence of integrons in the fecal microbiota at early stages of life and further suggest that the commensal gut microbiota can serve as a reservoir for multidrug resistance, potentially contributing to its rapid spread.

## Additional Information

**How to cite this article**: Ravi, A. *et al.* The commensal infant gut meta-mobilome as a potential reservoir for persistent multidrug resistance integrons. *Sci. Rep.* **5**, 15317; doi: 10.1038/srep15317 (2015).

## Supplementary Material

Supplementary Information

## Figures and Tables

**Figure 1:**
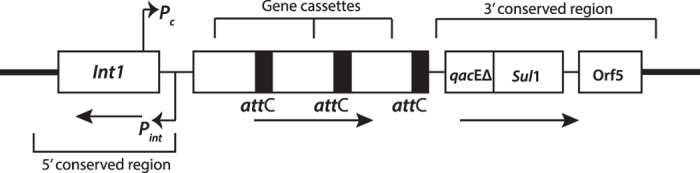
Structure of class I integron. A general representation of a class I integron with resistance gene cassettes at the attachment sites (*att*C) and a common promoter for the cassettes as *P*_*c*_ and for the integrase as *P*_*int*_. The following cassettes are a part of the 3′ conserved region and not mobile: s*ul*1 gene encoding resistance to sulfonamides and *qac*E∆ encoding resistance to quaternary ammonium compounds.
