# Supplementary material for: The commensal infant gut meta-mobilome as a potential reservoir for persistent multidrug resistance integrons
Source: Sci Rep. 2015 Oct 28;5:15317. doi: 10.1038/srep15317 (PMC4623605; doi:10.1038/srep15317)
Supplement: Supplementary Information [file srep15317-s1.pdf]

The commensal infant gut meta-mobilome as a potential reservoir for persistent multidrug resistance integrons

Anuradha Ravi[1ǂ], Ekaterina Avershina[1ǂ], Steven L. Foley[2], Jane Ludvigsen[1], Ola Storrø[3], Torbjørn Øien[3], Roar Johnsen[3], Anne L. McCartney[4], Trine M. L'Abée-Lund[5] and Knut Rudi[1]*

Supplementary information

The commensal infant gut meta-mobilome as a potential reservoir for persistent multidrug resistance integrons

Supplementary Table 1

Information on different factors for the mother-child pairs

Sample ID	**Mother asthma**	**Mother eczema**	**Mother allergy**
112			
119			
128	0	0	0
129			
136			
139			
148			
150	1	1	1
190	0	0	1
197	0	0	0
200	0	0	1
211	1	1	0
212			
217			
227	0	1	1
303			
306			
307	1	1	1
309	0	1	1
336	0	1	0
595	0	1	1
922	0	0	0
924	0	0	1
940			
1135	0	0	0
1143	0	1	1
1144	0	1	0
1226			
1233	0	0	0
1235	0	1	0
1244	0	0	0
1278	0	0	1
1299	0	0	1
1307			
1313	0	0	0
1321	0	0	0
1337	0	0	0
1345	0	1	1
1347	0	1	1
1356			

1361	1	1	1
1367			
1369	1	0	1
1373	0	1	1
1376	0	1	0
1379			
1380	0	0	1
1382	0	0	1
1383	1	0	1
1389			
1392	0	0	0
1419			
1424			
1437	1	1	1
1454			
1467	0	1	1
1473			
1479			
1485	0	0	1
1497	0	1	1
1515	0	0	0
1519			
1520	0	0	1
1521	0	0	0
1522			
1553			
1555	0	0	0
1563	0	0	0
1566	0	0	0
1612			
1625	0	1	1
1635	0	0	1
1636	0	0	1
1642			
1643	0	0	0
1645	1	1	1
1647	0	0	1
1648			
1649	1	0	0
1655	0	1	0
1659	0	1	1
1710	0	1	1
1715	1	1	1
1824	0	0	1
1852	1	0	1
1875	0	1	0
1882	0	0	0
1886	0	0	1
1899	0	0	1
1902	1	0	1

1905	0	0	0
1908	0	0	1
1909	0	1	1
1913			
1926			
1936			
1944	0	1	0
1946			
1951			
1958			
1965			
1995	0	1	1
2033	0	0	1
2040	0	0	1
2043	0	0	0
2048			
2050	0	0	0
2057			
2058	0	1	0
2062	0	0	0
2078	0	0	0
2088	0	0	0
2150	0	0	0
2161	0	1	0
2168			
2170	0	0	0
2213	0	0	0
2214	0	0	0
2223	0	0	0
2225			
2227			
2228			
2229	0	0	0
2243			
2244	0	0	0
2256			
2423	0	0	1
2449			
2503	0	1	0
2542	0	0	0
2549	0	0	1
2551	1	0	1
2553			
2556	0	0	0
2558			
2565	0	0	1
2573			
2591	0	0	0
2594	0	1	0
2622	0	1	1

2627	1	1	1
2632	0	0	1
20072	0	0	1
20074			
20077	0	0	0
20083			
20084	1	1	0

Collective information on allergy-related diseases, atopy, antibiotic usage; health and exposure factors

The commensal infant gut meta-mobilome as a potential reservoir for persistent multidrug resistance integrons

Father asthma	father eczema	Father allergy	Dampness index	ventilated more than once per day	Bleeding during pregnancy	formula for baby? (6w)	baby ear infection (1y)
			1	0	0	0	0
				0	0	0	1
1	1	1	0	0			0
			0	1	0	0	
			1	0	0	0	
			1	0	0	0	0
					0	0	0
0	0	0		1	1	1	1
0	0	1	1	0			
0	0	0	0	0	0	0	0
0	0	0	0	0	0	0	0
0	1	1	1	0	0	0	0
			0	0	0	0	0
			0	0	0	0	0
0	0	0	1	0			0
			0	0			0
				0			
0	0	0		1			
0	0	0	1	1	0	0	
0	0	1		1	0	0	
0	0	0		1	1	0	1
0	1	0	1	1			
0	0	0	0	0			0
			0	0	0	1	
0	0	1	0	1	0	0	0
1	1	1	0	0	0	0	0
0	0	1	0	1	1	0	0
				0	0	0	
0	0	1	0	0	0	0	1
0	1	0	0	0	0	1	0
0	1	0	1	0			
0	1	0	1	0			
0	1	0		1			
			0	0	0	0	0
0	0	0	0	0	1	0	0
0	0	1	0	1	0	0	0
0	0	0	1	0	0	0	
0	0	0	1	1	0	0	0
0	1	1		0	0	0	1
			1	0	0	1	0

0	0	0	1	0	1	0	0
			1	0	0	0	0
0	0	1	0	0	0	0	
0	0	0	1	0	0	0	0
0	0	1	1	0	0	0	0
			0	0	0	1	0
0	0	0	1	1	0	0	
0	1	0	0	0	0	0	
0	0	1	0	0			
			0	1	0	0	1
0	1	0	0	1	0	0	0
				0	0	0	0
			0	1	0	1	1
0	0	0	0	0			
			0	0	0	0	0
0	0	0	1	0	0	0	1
			0	1	0	0	0
			1	0			0
0	0	1	1	0	0	0	0
0	0	0	1	0	0	0	0
1	0	1		0		0	0
				0	0	1	1
0	0	0	0	0			
0	0	1	1	1	0	0	0
			0	0	0	0	0
					0	1	0
0	0	1	0	0	0	0	
1	1	0		0	1	0	1
1	0	1	0	1			0
			1	0	0	0	0
0	0	1		0	0	0	
0	0	1	1	0	0	1	0
0	0	1	0	0	1	0	0
			0	1	0	0	0
0	0	0	0	1	0	0	0
0	0	1	1	1	0	0	0
0	0	1	0	1	0	0	0
			0	0			
0	0	0	0	0			0
0	1	0	0	0	0	0	1
0	0	0	0	0			
0	0	0	1	0	0	1	
0	0	0	1	1	1	0	
0	0	0	0	0			0
0	1	0	0	1	1	0	
0	0	0		0	0	0	
0	0	0	1	0			1
0	0	0	0	0	1	0	
0	1	0	1	0	0	0	0
0	0	0	1	0			

0	1	1		0	0	1	0
0	0	0		0	0	0	0
1	0	0	1	0	0	0	0
			0	1	0	1	0
			0	0		0	
			0	0	0	0	0
0	0	1	0	0	0	0	0
			1	0	0	0	0
			0	0			
			0	0	0	0	1
1	0	1	1	0	0	0	0
0	0	1	1	0	0	0	
0	0	1		0	0	0	1
0	0	1	1	1	0	0	
			1	0	0	1	1
0	1	1	0	0			
			0	0			
0	0	1	0	0	0	0	0
0	1	0		0	0	1	0
0	1	1	1	0	0	0	0
0	0	1	0	0			
1	0	1	0	0	0	0	0
0	1	0	0	0	0	0	
			1	0		0	0
1	0	1	0	0	0	0	0
1	1	1	0	0	0	0	0
1	0	0		0			
0	0	1	0	0	0	0	0
					0	0	0
				0	0	1	0
				0	0	0	1
0	0	1	0	1			0
					0	0	0
0	0	0	0	0			
					0	0	0
0	0	0	1	0			
			1	0			
0	0	0	1	0	0	0	0
0	0	1	1	0		0	
0	0	1	1	0	0	0	0
0	0	1	1	0	1	0	0
				0	0	0	
1	1	0	0	0	0	0	0
			0	1	0	0	0
0	0	0		0	0	0	0
			0	0	0	0	
0	0	1	1	0	0	0	0
0	0	0	1	0			
0	0	0	0	0	0	0	

0	0	0		0			
0	0	0	1	1	0	0	1
0	1	0	0	0			
			1	0	0	0	0
0	0	1	0	0	0	0	
			1	1			
0	0	1	0	1	0	0	

for the selected mother and child pairs. From column B to N, 1 is yes and 0 is no and from column O to X, numb

The commensal infant gut meta-mobilome as a potential reservoir for persistent multidrug resistance integrons

treated with antibiotics?	formula for baby? (1y)	rice first time (months)	corn first time (months)	wheat first time (months)	bread first time (months)	cooked veg first time (months)
0	0			3	6	11
1	0			6	7	8
0	0	6	6	8	10	10
	0	4	4		9	7
0	1		5	7	9	6
1	1	10	10	10	13	13
0	0	5	6	6	10	6
0	0			4	9	4
	1		6	7	8	8
	0	9	4	4	6	6
	0			6	10	12
	1	5	4	5	7	7
0	0	3	3	64	6	6
1	1		4	5	7	6
	0	7	7	9	9	9
	0	4	4	4	6	7
0	0	6	6	6	8	6
	1		6	8	8	6
	1	4	4	5	7	6
1	0	4	4	5	8	9
0	0	10	6	6	10	11
	0	6		8	7	6
	0	6	6	7	9	9
	1		5	5	10	5
	0	5	5	7	7	6
1	0		4	5	10	9
	0	8		6	5	4

The commensal infant gut meta-mobilome as a potential reservoir for persistent multidrug resistance integrons

0	0	6	6		7	7
0	1	6		6	8	10
	0	6	5	5	5	6
0	1	4	4	5	10	11
0	1		3	3	10	6
	1	12		5	8	11
	1	6	6	6	9	8
	0	6	6	6	6	6
1	1	4	4	4	8	8
	0	5	5	6	7	9
1	0		5	6	8	6
	0	8	8	8	10	9
	0	4	4	5	5	6
	0	4	4	5	10	6
	1			6	10	6
1	1	4	4	5	7	7
0	0		4	6	10	6
0	0	5	5	6	7	8
	0	4	4	6	10	6
0	0			4	9	10
	1	6	6	6	6	6
0	1		4	6	8	6
0	1	4	6	6	10	10
	0	4	4	4	9	5
	1		4	5	6	6
0	0		7	7	8	8
0	0	5			8	4
0	0	6	6		9	9
	1	4	4	5	6	5
1	1	5		6	11	11
0	0	6	6	7	10	6
0	0		6	6	10	10
	0		6	6	6	6

	1	4	4	5	9	6
	1		4	5	7	7
	0	5	6	7	5	6
	0		4	6	6	
	0	6	6	9	8	10
0	0				9	9
	0		6	6	9	8
1	0	6	6	6	8	8
0	1	3	13	3	7	11
	0		4	6	9	7
0	0	4	4	6	8	4
0	0	5		8	9	9
	1	4	4	5	6	6
	0	4	4	8	6	10
	0			6	7	6
	0	11	5	8	9	7
0	1			6	9	9
0	0			6	8	8
	1		5	6	9	5
	0		4	4	9	6
	0	12		6	10	11
1	1			5	5	5
0	1	64	4	5	6	6
	0		5	7	6	6
0	0	5	5	9	7	9
	1	4	4	6	8	10
0	1	12	6		5	5
0	1	5	5	8	8	5
	1	4		6	6	6
	0		6	6	6	8
	1		4	6	6	6
0	0	10		7	7	7

The commensal infant gut meta-mobilome as a potential reservoir for persistent multidrug resistance integrons

0	1	6	6	6	8	6
0	1			9	9	9

ɔer represent the months.

raw veg first time (months)	fruits first time (months)	fish first time (months)	milk first time (months)	eggs first time (months)
	7	10	6	10
12	10	6	8	0
	10	12	11	
	7	12	12	
	7	9		
	15			
12	12	6	12	16
	6			
	6		12	12
8	7	5	8	
	6	12	12	
0	10	9	10	
10	6	7	11	12
10	5	7		
	6			
7	7	8	11	10
10	6	9		
8	6	10	12	12
	6		11	10
	5	12	11	
11	10	10	11	
8	8	10	10	
10	8		12	12
	4	5	12	6
	9	13	12	12
10	6	11	11	10
12	4	6	6	5

9	6			
	8			
7	6	8	11	
	8		11	
	6		12	
	9	11		
	6	9		11
6	6	6	5	6
7	9	9	7	
10	5	11	11	
11	6	10	12	12
	10			
6	6	9	9	12
	6	12	12	12
	6	8	11	
12	8	10	12	0
	6	10	12	10
	5	10		
6	4	10	12	
17	11		10	11
	6	6	12	
	6	6		
8	10	12	12	
10	10	11	10	
9	6	12	12	12
	8	11	11	9
11	4		11	
	7	12	12	10
8	5	11	12	
	7	12	12	12
9	6	8	12	70
10	6			
	6	6	11	

	4	12	12	
9	6			
12	5	8	12	12
	4	10	8	6
	6			
6	6	9	11	
11	9	12	11	
10	8	11	12	12
	6	11	11	12
16	7	12	12	12
11	6	10	12	12
10	9	12	10	
10	4	6		
10	6	11	12	
10	7	11	11	11
11	6			
	8	9	12	
	10		11	
	5	6	11	
12	6	6	10	
	6		7	
5	6	6	13	5
	7	9	11	
8	6	0	11	
10	6	10	11	
	8	12	12	12
	5	5	11	
10	6	10	11	12
18	6	12	12	12
8	6	8	12	12
10	9	11	12	12
9	6	8	10	

The commensal infant gut meta-mobilome as a potential reservoir for persistent multidrug resistance integrons

11	6	6		
	9	9	9	

Commensal infant gut meta-mobilome as a potential reservoir for persistent multidrug resistance integrons

Comparison of variance between triplicates

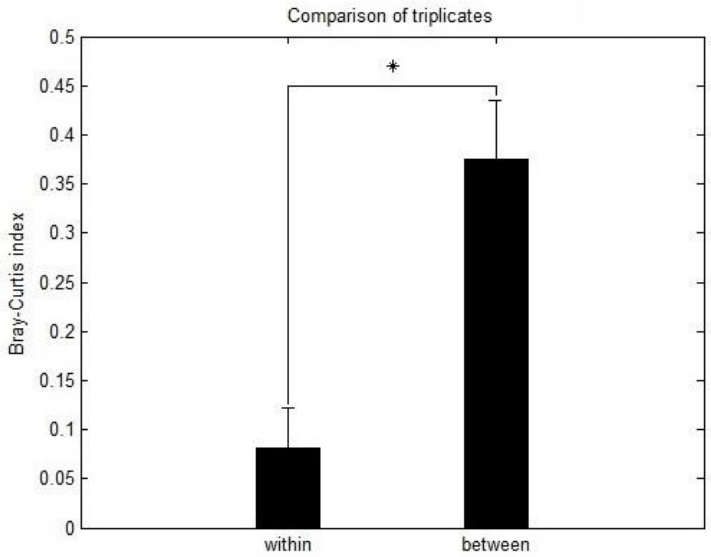

Supplementary fig. S1: Comparison of variance inside and between triplicates from the 16S metagenome illumina sequencing using Bray-Curtis dissimilarity analysis.

Commensal infant gut meta-mobilome as a potential reservoir for persistent multidrug resistance integrons

Correlations between OTU quantification and *int*1 gene

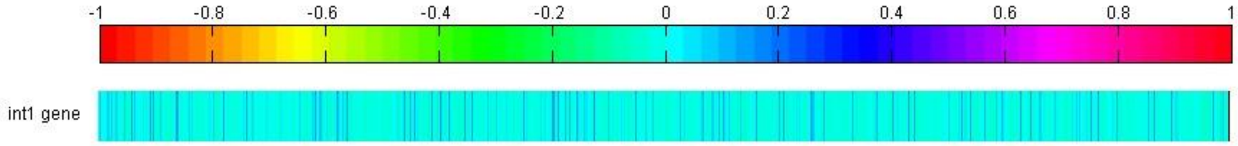

Supplementary Fig. S2: Correlation between OTU and *int*1 gene abundance

Commensal infant gut meta-mobilome as a potential reservoir for multidrug resistance integrons

Pairwise correlations between bacteria classes and *int*1 abundance

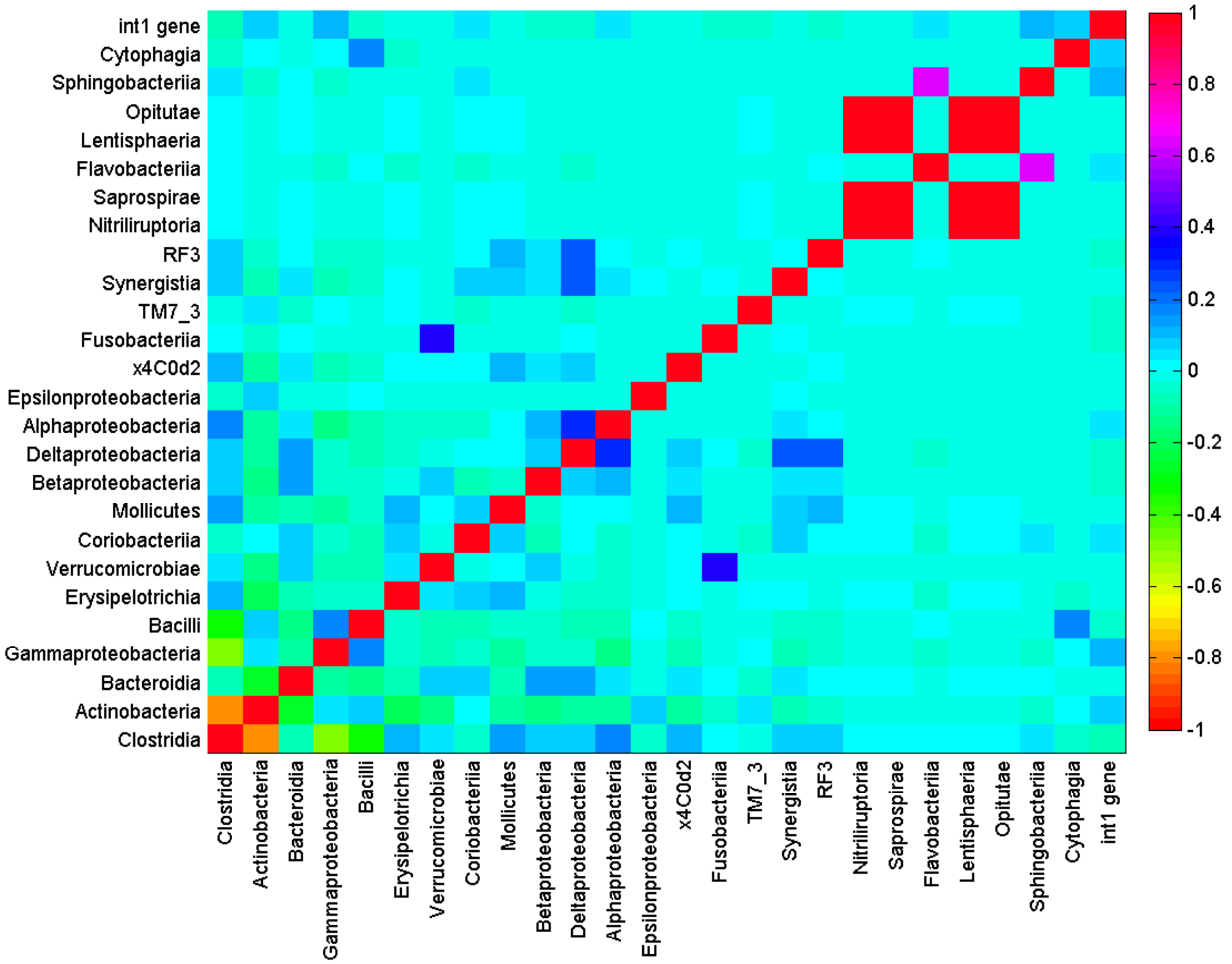

Supplementary fig. S3: Pairwise correlations of different bacterial classes and the abundance of *int*1 in the samples

Commensal infant gut meta-mobilome as a potential reservoir for persistent multidrug resistance integrons

Regression decision trees

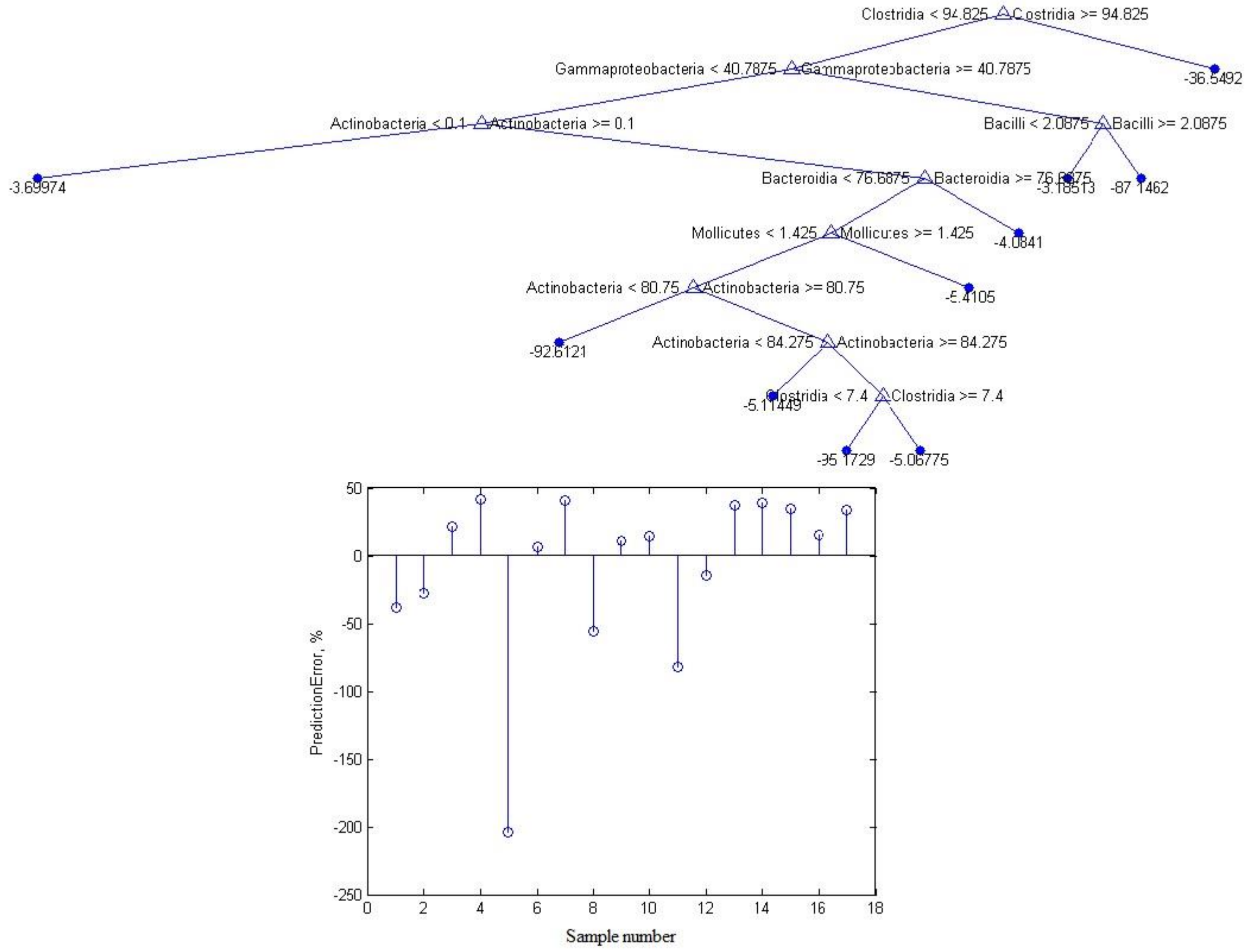

Supplementary fig. S4: Regression decision tree of predicting *int1* gene abundance based on bacterial class abundance

Commensal infant gut meta-mobilome as a potential reservoir for persistent multidrug resistance integrons

Classification desicion trees

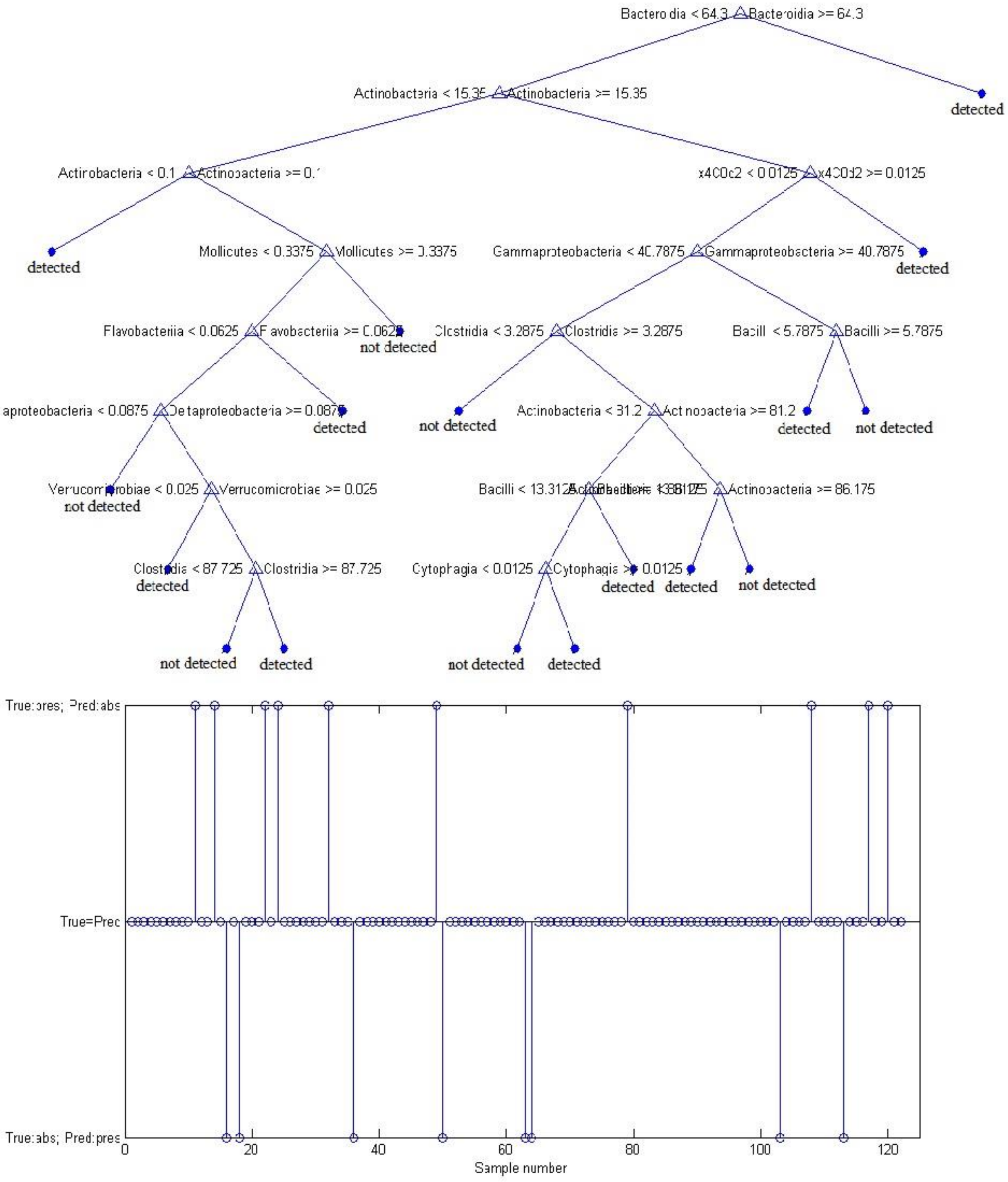

Supplementary Fig. S5: Classification decision tree of predicting *int1* gene detection based on bacterial class abundance

Commensal infant gut meta-mobilome as a potential reservoir for persistent multidrug resistance integrons

Graphical representation of reads mapping to plasmid pSH1148_107

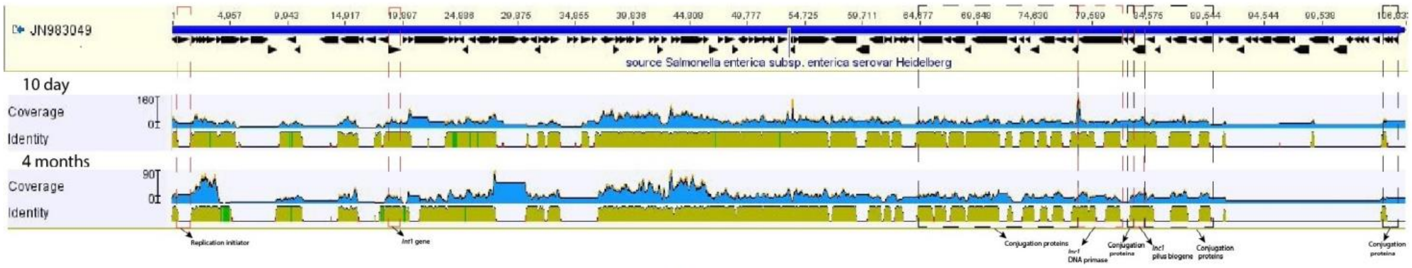

Supplementary fig. S6: Metagenomic reads mapping the integron-containing plasmid. Structural features: Dark blue triangles- coding directions of the genes, red triangle-coding region of *int1* gene, blue- coverage of the reads at 3-10 days and 4 months, green- identity between the reads and plasmid and dark green patches- regions of high identity.
